# Nuclear location of tumor suppressor protein maspin inhibits proliferation of breast cancer cells without affecting proliferation of normal epithelial cells

**DOI:** 10.1186/1471-2407-14-142

**Published:** 2014-02-28

**Authors:** Magdalena Machowska, Katarzyna Wachowicz, Mirosław Sopel, Ryszard Rzepecki

**Affiliations:** 1 Laboratory of Nuclear Proteins, Faculty of Biotechnology, University of Wroclaw, 63/77 Przybyszewskiego Street, 51-148 Wrocław, Poland; 2 Department of Biology and Botanical Pharmacy, Medical University, 211 Borowska Street, 50-556 Wrocław, Poland; 3 Present address: Division of Translational Cell Genetics, Department for Pharmacology and Genetics, Medical University of Innsbruck, 1a Peter Mayr Street, A-6020 Innsbruck, Austria

**Keywords:** Maspin, Breast cancer, Nuclear maspin, Cell proliferation, Ki-67

## Abstract

**Background:**

Maspin, which is classified as a tumor suppressor protein, is downregulated in many types of cancer. Several studies have suggested potential anti-proliferative activity of maspin as well as sensitizing activity of maspin for therapeutic cytotoxic agents in breast cancer tissue culture and animal models. All of the experimental data gathered so far have been based on studies with maspin localized cytoplasmically, while maspin in breast cancer tumor cells may be located in the cytoplasm, nucleus or both. In this study, the effect of maspin cytoplasmic and nuclear location and expression level on breast cancer proliferation and patient survival was studied.

**Methods:**

Tissue sections from 166 patients with invasive ductal breast cancer were stained by immunohistochemistry for maspin and Ki-67 protein. The localization and expression level of maspin were correlated with estimated patient overall survival and percent of Ki-67-positive cells. In further studies, we created constructs for transient transfection of maspin into breast cancer cells with targeted cytoplasmic and nuclear location. We analyzed the effect of maspin location in normal epithelial cell line MCF10A and three breast cancer cell lines - MCF-7, MDA-MB-231 and SKBR-3 - by immunofluorescence and proliferation assay.

**Results:**

We observed a strong positive correlation between moderate and high nuclear maspin level and survival of patients. Moreover, a statistically significant negative relationship was observed between nuclear maspin and Ki-67 expression in patients with invasive ductal breast cancer. Spearman’s correlation analysis showed a negative correlation between level of maspin localized in nucleus and percentage of Ki-67 positive cells. No such differences were observed in cells with cytoplasmic maspin. We found a strong correlation between nuclear maspin and loss of Ki-67 protein in breast cancer cell lines, while there was no effect in normal epithelial cells from breast. The anti-proliferative effect of nuclear maspin on breast cancer cells was statistically significant in comparison to cytoplasmic maspin.

**Conclusions:**

Our results suggest that nuclear maspin localization may be a prognostic factor in breast cancer and may have a strong therapeutic potential in gene therapy. Moreover, these data provide a new insight into the role of cytoplasmic and nuclear fractions of maspin in breast cancer.

## Background

Maspin (Mammary Serine Protease Inhibitor) was first identified in normal mammary glands and breast cancer cells. Based on structural homology, maspin belongs to the serpin superfamily (serpin b5) [1]. Since serpin b5 has no inhibitory activity against serine proteases, its role in cells is not fully understood [2].

In normal mammary glands, maspin is expressed, at a high level, in myoepithelial cells, while it is not found in luminal cells [1,3]. Maspin may be located in cytoplasm, nucleus, at the cell surface and in extracellular matrix of myoepithelial cells in normal mammary glands [4]. However, there have been no specific domains or sequences identified that may ensure maspin nuclear localization or secretion. Maspin is responsible for cell adhesion and mobility during embryogenesis and mammary gland development [5]. Maspin is also important in the development of the mammary gland. At the early stage of lactation, maspin causes a lower level of milk proteins, including casein and WAP (whey acidic protein) [6].

Maspin is classified as a class II tumor suppressor. Maspin demonstrates proapoptotic, antimetastatic and antiangiogenic properties, exerting an inhibitory effect on tumor cell survival, mobility, invasiveness and metastasis ability, and also reduces the tumor tissue vascularization [7-10]. In many studies, it has been found that a decreased level of maspin causes cancer progression and transition from non-invasive to invasive cancer [11,12]. *In vitro* studies showed that in primary cell lines derived from tumors maspin is expressed, while after several passages the maspin level decreases until complete loss [7,13]. In secondary breast cancer cell lines maspin is absent [14]. Clinical data indicate a positive correlation between higher maspin expression level and lower degree of differentiation, lower grade of tumor and improved survival of patients [10,15].

Despite these data, there are some controversial and contradictory data about maspin prognostic significance and importance of its expression. In many cancer studies, including those related to breast cancer, a negative and positive correlation are described with reference to high or low maspin expression level as a prognostic factor of tumor development [16-19].

Many reports have suggested that biological significance, activity and clinical implications of maspin in various types of cancer depend on its subcellular localization [19-22]. In many types of cancers, including breast, ovarian, lung, larynx, renal and colon cancer, there has been indicated a positive correlation between nuclear maspin location and molecular markers of good prognosis, benign instead of malignant form of cancer, better patient survival and long-term remission [19,20,23-26]. However, the significance of nuclear maspin localization in cancer is still not clear enough to use maspin localization pattern as an unquestioned diagnostic or prognostic factor. Maspin’s mechanism of action, especially its nuclear fraction, is not very well understood and requires further examination for better understanding. Recently, a few attempts have been made to clarify this controversy of anticancer activity and molecular mechanism of action of maspin using different models [22,27-29] but they have not clarified fully the essential question of the potential different activities of cytoplasmic and nuclear fraction of maspin, because in studies performed so far maspin was mainly localized in cytoplasm or ubiquitously in cytoplasm and cell nucleus [22,30,31].

That is why we made an attempt to develop a breast cancer tissue culture model system for studies of function of cytoplasmic and nuclear maspin independently. This breast cancer cell line model system together with clinical data from the patients allowed us to resolve the effect of nuclear and cytoplasmic maspin in breast cancer on proliferation and its potential as a genetic drug in breast cancer gene therapy.

## Methods

### Patient samples and ethical issues

**Figure 1:**
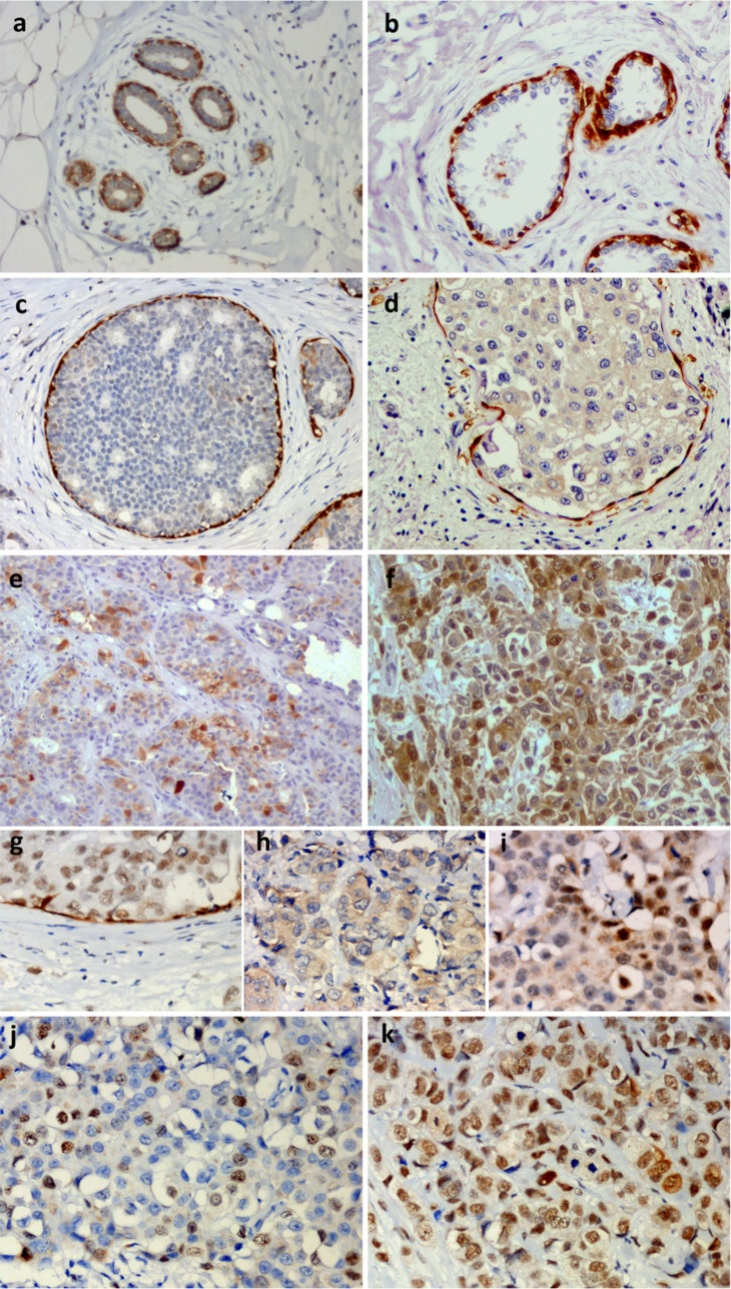
**Immunohistochemical staining of breast cancer specimens from patients.** Staining for: maspin **(a-i)** and Ki-67 **(j, k)**; a - maspin localization in normal human mammary gland; **b-f** - maspin localization in different stages of breast cancer, as follows: early stage of ductal breast cancer, ductal carcinoma in situ, early stage of invasive breast cancer, invasive breast cancer **(e, f)**; **g-i** - subcellular maspin localization in breast cancer, as follows: nuclear, cytoplasmic, mixed; **j** - low grading tumor with low percentage of Ki-67 positive cells; **k** - breast cancer with high percentage of Ki-67 positive cells in malignant cancer.

Breast tumor tissue sections for statistical analysis were taken intraoperatively from 166 women diagnosed with invasive ductal breast cancer. For visualization of maspin location during cancerogenesis (see Figure 1) breast tumor sections were stained also from material taken from women diagnosed with other stages of cancer: early stage of ductal breast cancer, ductal carcinoma in situ, early stage of invasive breast cancer. Slices of breast tumor tissue were collected in accordance and with the recommendations of the: “Bioethical Committee of the Lower Silesian Oncology Center”. Research was performed on archived, fixed and paraffin-embedded breast tissue specimens obtained during breast cancer surgery. According to Polish regulations we do not need to obtain patients consent for studies on archived tissue specimens. Sample processing, data processing and analyses were performed in full compliance with all bioethical regulations and in accordance with Polish law. Bioethical Committee of the Lower Silesian Oncology Center approved the study.

4% formalin-fixed and paraffin-embedded samples for histopathological examination were routinely stained with hematoxylin and eosin. Histological evaluation of specimens was made by two independent pathologists. The characteristics of the patients are shown in Tables 1. Adjacent slices of the same paraffin blocks were used for immunohistochemistry.

**Table 1:** Patients’ characteristics N = 166

**Characteristic**	**N**	**%**
**Age**		
< = 50	22	13.3
51-60	42	25.3
61-70	57	34.3
> 70	45	27.1
**Tumor (T)**		
T1	68	41
T2	40	24.1
T3	34	20.5
T4	24	14.4
**Lymph nodes (N)**		
N0	59	35.5
N1	107	64.5
**Metastasis (M)**		
M0	124	74.7
M1	42	25.3
**Grade (G)**		
G1	57	34.4
G2	54	32.5
G3	55	33.1

### Immunohistochemistry

Paraffin sections were placed on Superfrost + slides, deparaffinized in xylene, rehydrated in a series of decreasing concentrations of ethanol and washed in phosphate buffered saline (PBS). After antigen retrieval by boiling in a microwave in citrate buffer (pH 6.0), the endogenous peroxidase activity was blocked by incubating sections with 3% H_2_O_2_ solution for 30 minutes.

Slices were incubated for one hour at room temperature with primary antibodies diluted in background reducing buffer (DakoCytomation): mouse anti-human maspin (clone G167-7, BD Pharmingen), dilution 1:400 or mouse anti-Ki-67 (clone MIB-1, DakoCytomation), dilution 1:10. After washing three times with PBS for 5 minutes, slices were incubated for 15 min with secondary, biotinylated antibody, followed by 30 min incubation with streptavidin-horseradish peroxidase (DakoCytomation). Color was developed with 3,′-diaminobenzidine (DAB) for 3-7 minutes. Nuclei were stained with Mayer’s hematoxylin.

To evaluate maspin expression, a semiquantitative method [32] was used to determine reaction intensity and percentage of positive cells from three representative fields of the specimen at 40× magnification. Specimens were scored according to percentage: 1-5% - 1 point, 6-50% - 2 points, >50% - 3 points and reaction intensity: weak - 1 point, moderate - 2 points, strong - 3 points. Points for both percentage and intensity were added and specimens were categorized into four groups: negative reaction - 0 points (0 points for percentage and intensity), weak reaction - 1 point (1-2 points), moderate reaction - 2 points (3-4 points), strong reaction - 3 points (5-6 points). Evaluation of maspin expression was conducted independently for the cytoplasm and the nucleus using the same procedure.

To evaluate the proliferative status, three representative fields with the typical signal from Ki-67 antigen was chosen [33]. At 40× magnification, nuclei showing expression of Ki-67 antigen in relation to all nuclei were counted. Proliferative status of tumors was evaluated as the percentage of positive cells and classified into two groups: less than 20% and more than 20% (according to the limit value of predictive and prognostic assessment of Ki-67 expression in breast cancer).

### Cell culture

Human breast cancer cell line MCF-7 was cultured in Eagle’s medium (EMEM, Lonza) and MDA-MB-231 and SKBR-3 cell lines were cultured on Dulbecco’s modified Eagle’s medium (DMEM with low glucose, Lonza) supplemented with 10% fetal bovine serum (Sigma), 1% Glutamax (Gibco) and 1% antibiotic/antimycotic (Gibco). MCF10A cell line was cultured on DMEM/F12 medium (medium mixture, Lonza) supplemented with 5% horse serum (Gibco), 1% Glutamax (Gibco), 1% antibiotic/antimycotic (Gibco), 20 ng/ml epidermal growth factor (Sigma), 0.5 µg/ml hydrocortisone (Sigma), 100 ng/ml cholera toxin (Sigma) and 10 µg/ml insulin (Sigma). MCF-7 (ATTC HTB-22), SKBR-3 (ATTC HTB-30), MDA-MB-231 (ATTC HTB-26) and MCF10A (ATTC CRL-10317) cell lines were kindly provided by Dr Paweł Surowiak (Wrocław Medical University) and Prof. Hermann Lage (Institute of Pathology, Charite Campus Mitte, Humboldt University, Berlin). MCF-7 stable cell lines expressing enhanced green fluorescent protein (EGFP) and maspin-EGFP were cultured in supplemented Eagle’s medium with G418 antibiotic in a final concentration of 150 µg/ml. All cell lines were maintained at 37°C in 5% CO_2_.

### Plasmid construction

Control plasmid pEGFP-C1 ensuring expression of EGFP was purchased from Clontech. Expression vector with maspin cDNA sequence (NM_002639) was constructed based on pReceiver M03 (GeneCopoeia). In order to obtain nuclear localization of maspin with fusion protein EGFP, the nuclear localization signal (NLS) was inserted between the sequences encoding maspin and EGFP. NLS was inserted by site-directed mutagenesis using QuikChange XL kit (Stratagene). Sequences of primers are the following: (forward/reverse) 5′-*CTCCTTACGGTCAT***CCAAAAAAGAAGAGAAAGGTC***ATGGCTAGCGTGAGC*-3′; 5′-*GCTCACGCTAGCCAT***GACCTTTCTCTTCTTTTTTGG***ATGACCGTAAGGAG*-3′. The amino acid sequence of NLS is PKKKRKV.

### Stable and transient cell transfection

For generation of stable cell lines expressing EGFP, maspin-EGFP and maspin-NLS-EGFP, MCF-7 cells were plated on a 10 cm cell culture dish and after 24 hours transfected with complexes of plasmid DNA and MetafectenePro (Biontex). Six hours later cell culture medium was removed and replaced with complete medium and after 48 hours the post-transfection medium was replaced with complete selection medium containing antibiotic G418 to a final concentration of 400 µg/ml. The selection was monitored by EGFP expression and negative cell death and G418 concentration was gradually reduced.

For transient transfection to immunofluorescence analysis breast cancer cells were plated on a 24-well plate 24 hours before transfection. Six hours after transfection with complexes of MetafectenePro and plasmid DNA medium was replaced with fresh complete medium. After appropriate time cells were fixed and stained as described below.

### Immunofluorescence

For immunofluorescence experiments, cells were plated on glass coverslips, fixed in 4% paraformaldehyde for 20 minutes and permeabilized with 0.5% Triton X-100 in PBS. Fixed cells were incubated in primary antibody solution overnight at 4°C, washed with PBS, and incubated for 1 hour with secondary antibody solution at room temperature and washed again in PBS. Coverslips were mounted on glass slides using DABCO mounting medium (Fluka) with DAPI. Staining was visualized on a Zeiss 510 Meta confocal microscope using the 63X objective. Primary antibodies used for staining were mouse anti-human maspin (BD Pharmingen, 1:25), mouse anti-human Ki-67 (DakoCytomation, 1:50), rabbit anti-human lamin C (a kind gift from prof. C.J Hutchison, 1:20). Secondary antibodies were donkey anti-mouse conjugated with TRITC and donkey anti-rabbit conjugated with Cy5 (both from Jackson ImmunoResearch). After 24 hours to 120 hours following transfection, EGFP-expressing cells were counted for expression of Ki-67 antigen in five to ten fields of view and the Ki-67-positive subpopulation was calculated as the percentage of all transfected cells.

### Cell proliferation assay

For cell proliferation assay cells were plated on a 10 cm cell culture dish and after 24 hours transfected with complexes of plasmid DNA and MetafectenePro (Biontex). Medium was replaced every three days with complete medium (without G418). After 24 hours to 9 days following transfection EGFP-expressing cells and all cells were counted in four fields of view and the EGFP-positive subpopulation was calculated as the percentage of all cells.

### Statistical analysis

SPSS software (SPSS version 17.0., Chicago, Illinois, USA) was used for statistical analysis of histopathological results. In order to analyze the correlation between the maspin protein level in cytoplasm and/or nucleus and the presence of Ki-67 antigen, contingency tables were used. Statistical significance of differences was assessed using the Pearson’s chi-square test or, if the assumptions were not met, Fisher’s exact test estimated with simulation methods. The correlation was evaluated by Spearman’s test. Significance was defined at the level of P-value ≤0.05 by the two-tailed test. Overall survival was estimated by the Kaplan-Meier method. The logrank test (the Mantel-Cox test) was used to evaluate statistical differences between two curves (for cytoplasmic pool of maspin and nuclear pool of maspin).

Microsoft Office Excel 2003 was used for statistical analysis of data obtained from experiments on cell lines. To evaluate the significance of differences between control and experimental groups the two-tailed Student’s *t* test was used.

## Results

### Maspin expression and localization in normal and cancer tissue from breast

In normal breast tissue, maspin is expressed in myoepithelial cells (Figure 1a). During cancer development, there is a decline in maspin level in myoepithelial cells and maspin protein is expressed in other cells (Figure 1b-f). Breast cancer cells showed nuclear, cytoplasmic or mixed maspin location (Figure 1g-i).In order to analyze the subcellular location and assess the level of expression of maspin in normal and tumor tissues of breast, a collection of tissue sections from 166 patients with invasive ductal breast cancer was studied using immunohistochemistry. The maspin-specific staining intensity in ductal breast cancer specimens was compared with staining of myoepithelial cells from regions of normal mammary tissue (Figure 1a) and cancer in situ specimens (Figure 1b,c). The staining intensity in myoepithelial cells was generally stronger than in breast cancer tissue.

The intensity and subcellular distribution of maspin protein were assessed and counted in specimens of breast cancer tissues. Positive staining for maspin was observed in 148 cases (89.2%) and lack of this protein in 18 cases (10.8%). In the majority of cases (N = 91; 54.8%) mixed nuclear and cytoplasmic maspin location was observed. In Tables 2 and 3, the distribution of maspin staining localization and intensity is shown. These data indicate that breast cancer specimens showed a heterogeneous level of maspin protein as well as various levels of maspin fraction in cytoplasm and nuclei and that this heterogeneity of maspin level and location should be taken into the future considerations and statistical analyses. Moderate and high levels of nuclear maspin correlate with better prognosis for patients.

**Table 2:** Maspin localization in patients’ specimens

**Maspin localization**	**Number of cases**
Mixed	91 (54.8%)
Cytoplasmic	22 (13.3%)
Nuclear	35 (21.1%)

**Table 3:** Maspin intensity of staining

**Maspin staining intensity**	**Cytoplasm**	**Nucleus**
Lack	54 (32.5%)	38 (22.9%)
Weak	27 (16.3%)	35 (21.1%)
Moderate	71 (42.8%)	84 (50.6%)
Strong	14 (8.4%)	9 (5.4%)

### Correlation between maspin expression level, location and proliferation status and prognosis for patients

**Figure 2:**
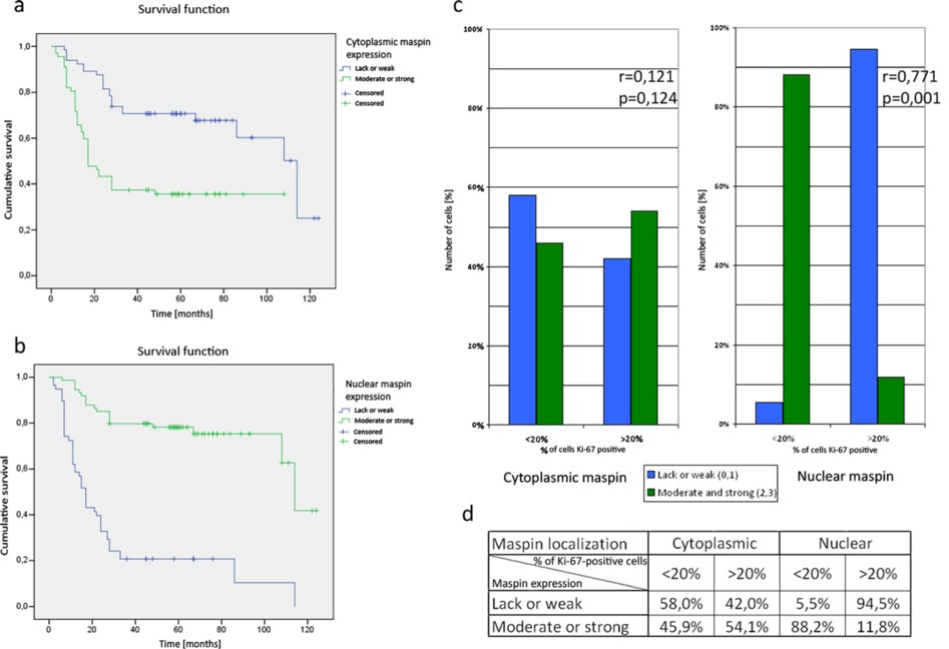
**Maspin influence on survival and proliferative status of cells from breast tumor specimens. a, b** - Kaplan-Meier analysis of survival of patients with breast cancer according to the localization and expression level of maspin; nuclear localization and higher level of this maspin pool positively affect the survival of patients unlike the cytoplasmically localized maspin and its higher level; **c, d** - correlation between proliferative status and maspin localization and expression level in breast cancer patients; strong and moderate level of nuclear maspin correlates with smaller subpopulation of cells expressing Ki-67 (less than 20%) in specimens from patients in contrast to weak expression or lack of nuclear maspin; there is no significant correlation between cytoplasmic maspin and Ki-67 expression; N = 166.

The data about level of maspin and amount of maspin in particular subcellular locations were analyzed with respect to patient survival rate as well as proliferation status of the cancer cells from patients. The analyses of expression of Ki-67 protein (typical proliferation marker used in cancer diagnosis) in particular cells were performed in parallel in order to assess the proliferation status of cancer cells (Figure 2c,d) [34,35]. Figure 1, sections j and k demonstrate low grading tumor and high grading tumor (according to Nottingham scale) with low and high percentage of Ki-67 positive cells respectively.For Kaplan-Meier analyses of survival of patients with breast cancer, samples were divided into two groups representing the subcellular status of maspin - cytoplasmic or nuclear - and the level of protein in particular fractions: low level or lack of protein and moderate and high level of protein (Figure 2a,b). Such organization of data allowed us to demonstrate the strong positive correlation between high nuclear maspin level and the survival of patients. Negative prognosis was associated with high level of cytoplasmic maspin while lack or low level of cytoplasmic maspin correlated with better prognosis than high cytoplasmic maspin.In order to analyze the correlation between maspin location and Ki-67 protein expression (proliferation status), specimens were divided and categorized into two groups: less than 20% Ki-67 positive cells (86, 51.8%) and more than 20% (80, 48.2%) (Figure 2c,d). No significant relationship (p = 0.118) was observed between cytoplasmic maspin and Ki-67 expression. Moreover, there was no significant correlation between cytoplasmic maspin and percentage of Ki-67 positive cells in the two groups (Spearman’s correlation r = 0.121; p = 0.124).

There was a statistically significant relationship observed between nuclear maspin and Ki-67 expression (p < 0.001). Spearman’s correlation analysis showed a negative correlation between level of maspin localized in nucleus and percentage of Ki-67 positive cells (r = 0.771; p < 0.001). In specimens in which less than 20% of cells expressed Ki-67, most cells showed moderate or strong staining intensity of nuclear maspin. Most cases of lack or weak staining intensity of nuclear maspin were observed in the second group - more than 20% of Ki-67 positive cells.

### Cell culture model for studies of maspin’s subcellular fraction anticancer activity

**Figure 3:**
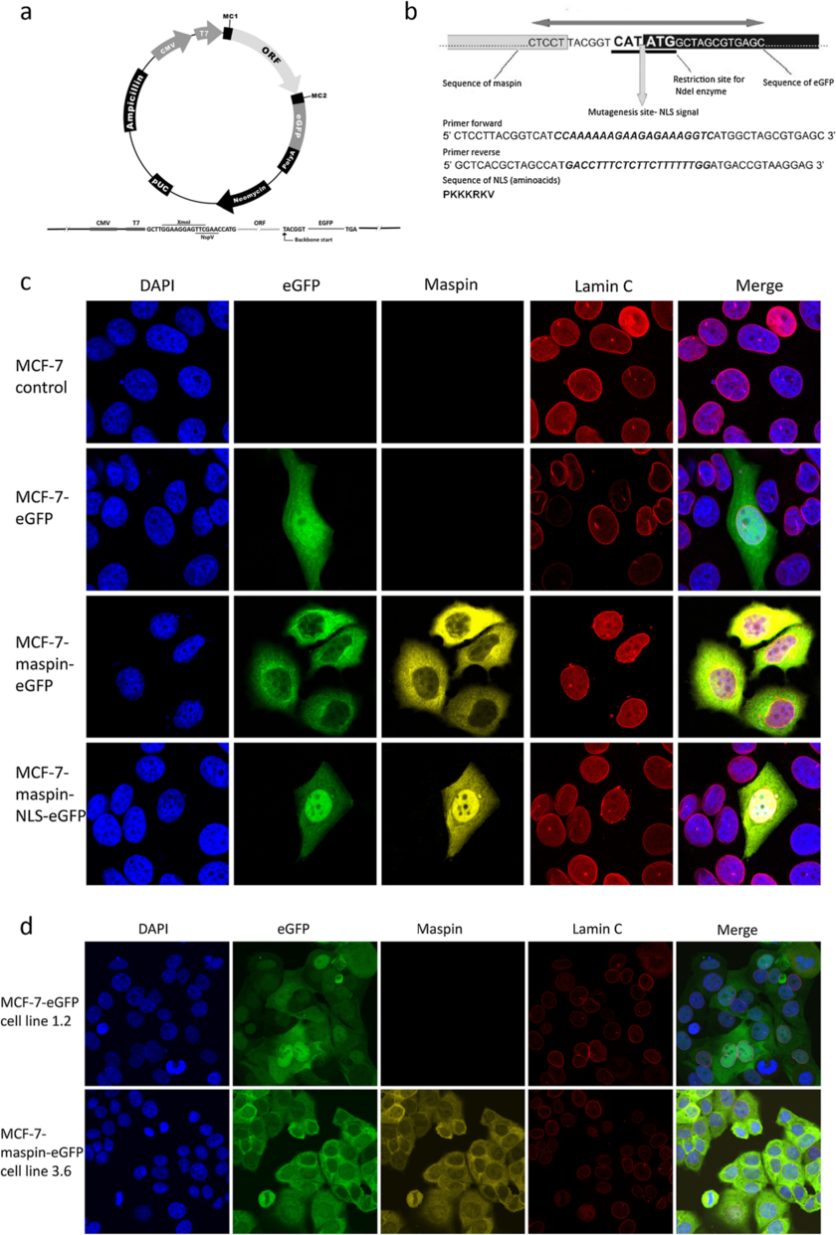
**A tissue culture model system for studies of location-dependent maspin’s effect on breast cancer cells. a** - pReceiver M03 plasmid map in which the sequence of maspin (NM_002639) was cloned; **b** - sequences of primers for mutagenesis to the nuclear localization signal (NLS) insertion; **c** - transient transfection of MCF-7 cells with three plasmids: pEGFP, Msp-EGFP and Msp-NLS-EGFP, maspin staining pattern confirms proper localization in transfected cells; **d** - stable cell lines: MCF-7 EGFP clone 1.2 expressing EGFP, MCF-7 maspin-EGFP clone 3.6 expressing maspin with fusion protein EGFP localized in cytoplasm; generation of cell line with nucleus-localized maspin failed because of death of all transfected cells.

Generation of cancer cells or cancer cell lines with maspin located in cytoplasm or nucleus seemed to be the best way to evaluate maspin localization influence on cell proliferation and to compare *in vitro* results to clinical data. Since the size and the structure of maspin theoretically allows maspin to passively diffuse through nuclear pore complexes (NPCs) (with all possible disadvantages of such diffusion taking place), we decided to use maspin fusion proteins with EGFP. We aimed at selecting MCF-7 breast cancer cell lines stably expressing maspin-EGFP fusion protein as a model for cytoplasmic maspin and maspin-NLS-EGFP fusion protein as a model for nuclear maspin. As an additional control we intended to select an EGFP protein expressing MCF-7 cell line. According to our expectations MCF-7 cells transfected with maspin-EGFP construct showed maspin-EGFP location exclusively in cytoplasm while cells transfected with maspin-NLS-EGFP construct showed nuclear accumulation of the protein (Figure 3c). A weak signal from cytoplasm from maspin-NLS-EGFP protein may result from newly synthesized protein not yet transported into the nucleus and/or actively stopped in the cytoplasm by interaction with other proteins. Cells transfected with EGFP protein showed mixed nuclear and cytoplasmic location of this protein (Figure 3c). All of our attempts to generate stable cell lines expressing maspin-NLS-EGFP protein failed. All cells died within 2-3 weeks time after transfection, while we had no problems with selection of many cell lines stably expressing maspin-EGFP protein and EGFP protein (Figure 3d). This suggested that nuclear location of maspin is toxic for MCF-7 breast cancer cells during longer periods of time while cytoplasmic maspin-bearing cells could survive and proliferate.

### Maspin location and proliferation of transfected MCF-7 cells

**Figure 4:**
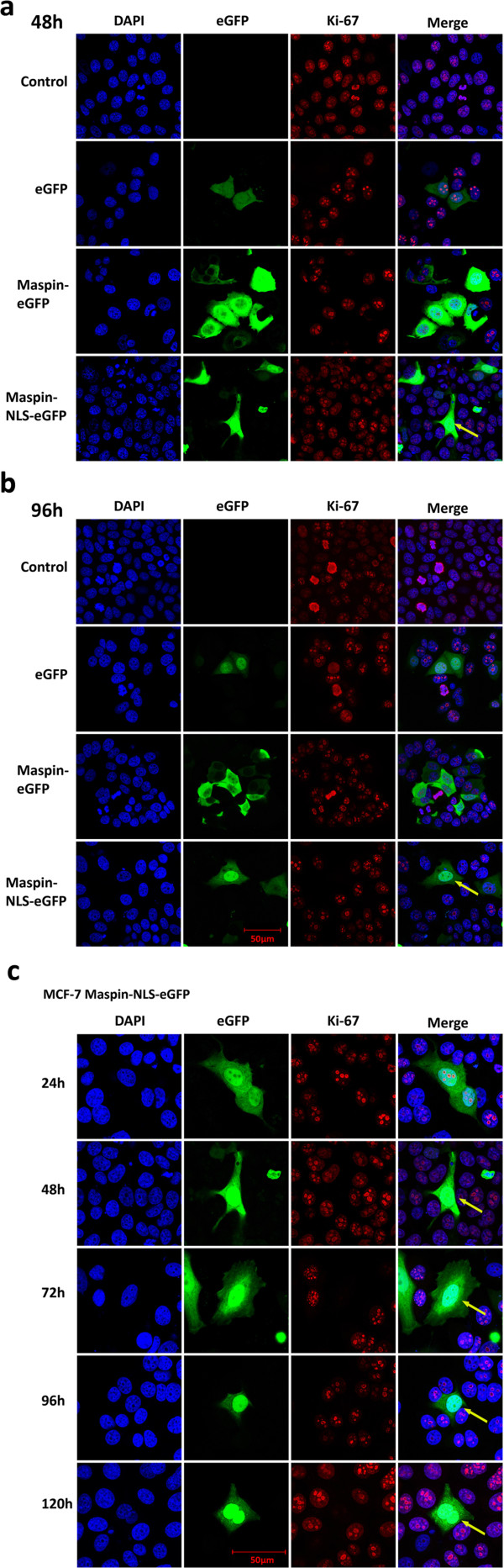
**Analyses of Ki-67 protein expression in MCF-7 cells with expression of EGFP, maspin-EGFP and maspin-NLS-EGFP.** Cells were transiently transfected with plasmids containing transgenes: EGFP, maspin-EGFP and maspin-NLS-EGFP. **a, b** - Cells fixed at 48 h and 96 h after transfection. **a** - 48 hours after transfection some cells expressing high levels of maspin localized in nucleus demonstrate reduction in Ki-67 protein; **b** - after 96 hours the subpopulation of cells with nuclear maspin expressing Ki-67 is decreased; at the same time there are no significant differences in control cells and cells transfected with EGFP and maspin-EGFP localized in cytoplasm; **c** - magnification of cells with nuclear localization of maspin at 24 to 120 hours after transfection and gradual loss of Ki-67 protein; arrows show cells expressing nuclear maspin that lost Ki-67 protein.

**Figure 5:**
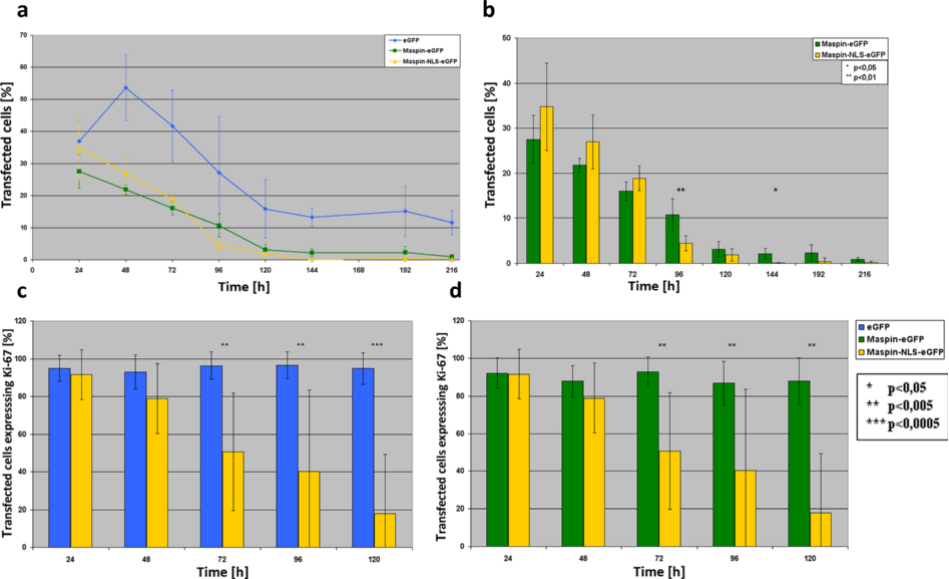
**Statistical analysis of cell proliferation and Ki-67 expression in transfected MCF-7 cells.** Control cells and cells transfected with three plasmids - EGFP, maspin-EGFP and maspin-NLS-EGFP - were subjected to statistical analyses of proliferation and Ki-67 protein expression; **a, b** - cell proliferation assay at 24 h to 9 days after transfection; only after 96 h and 144 h were there significant differences between cells expressing cytoplasmic and nuclear maspin, but generally cells in both transfections died rapidly; however, there were new colonies in MCF-7 msp-EGFP cells, in contrast to msp-NLS-EGFP cells; **c, d** - percent of transfected cells expressing Ki-67 protein; there are significant differences at 72 h after transfection between cells expressing nucleus-localized maspin and those expressing both cytoplasm-localized maspin and EGFP; these differences increase at 96 h and 120 h after transfection.

Since we failed to select stable cell line expressing maspin-NLS-EGFP we were forced to perform further experiments on transiently transfected cells. We decided to analyze the proliferation rate and phenotype of MCF-7 cells expressing EGFP protein, cytoplasmic maspin and nuclear maspin at various time points after transfection between 24 h and 120 h (1-5 days). Figure 4a,b demonstrates the typical phenotypes of MCF-7 cells transiently transfected with plasmids coding for EGFP, maspin-EGFP and maspin-NLS-EGFP proteins after 48 h and 96 h as the most essential for analyses. At the first time points (24 - 72 h) cells with cytoplasmic maspin and nuclear maspin showed similar phenotype with respect to size and shape (Figure 4a-48 h). At time points of 72 up to 120 h we started to observe a decrease in the number of cells expressing Ki-67 protein in MCF-7 cells with nuclear maspin (Figure 4b-96 h). Figure 4c summarizes the general trend observed in analyzed cells expressing nuclear maspin. Since Ki-67 protein is a marker of proliferation we analyzed the proliferation rate and performed statistical analyses of Ki-67 protein expression in transfected MCF-7 cells.Cell populations of the attached, transfected and non-transfected cells were daily counted for 9 days and then the percent of transfected cells in comparison to all cells was calculated (Figure 5a,b). The efficiency of transfection in all three cases was about 35% (after 24 hours). After 48 hours, there was an increase in percentage of EGFP transfected cells and a decrease in the maspin-EGFP and maspin-NLS-EGFP subpopulation. From 24 and 48 hours after transfection the percentage of cells expressing maspin-EGFP and maspin-NLS-EGFP decreased rapidly. The high increase of percentage of EGFP-expressing cells between 24 and 48 h was a result of increased efficiency of production of EGFP protein in transfected cells above the detection level and a lower level of overall transfection toxicity.Until the third day (up to 72 h) after transfection, a decrease of percent of transfected cells (for all plasmids) was a net result of cell death and higher proliferation rate of untransfected cells. After that time, new colonies expressing transfection marker (EGFP) appeared in EGFP and maspin-EGFP transfected cells. However, there was no new colony formation observed in maspin-NLS-EGFP cells. After the third day, a further decrease of percent of transfected cells was still mainly caused by a higher proliferation rate of non-transfected cells. Significant differences in proliferation were noticeable separately between EGFP and maspin-EGFP and EGFP and maspin-NLS-EGFP. Statistically significant differences between maspin-EGFP and maspin-NLS-EGFP were observed only at 96 h and 144 h after transfection (Figure 5b).We performed statistical analyses of the amount of cells expressing Ki-67 protein among MCF-7 cells expressing EGFP and cytoplasmic and nuclear maspin (Figure 5c,d). We found a strong correlation between nuclear maspin and disappearance of Ki-67 protein at time points starting from 72 h. No correlation was found for cytoplasmic maspin and EGFP protein. This indicates that nuclear maspin inhibits proliferation of MCF-7 breast cancer cell lines starting from 72 h by mechanisms triggering degradation or inhibition of expression of Ki-67 protein. This implies that only nuclear but not cytoplasmic maspin shows this anti-proliferative activity. These data also are in perfect agreement with the nuclear maspin prognostic marker for patients with breast cancer.

### Nuclear maspin shows its anti-proliferative activity in other breast cancer cell lines

**Figure 6:**
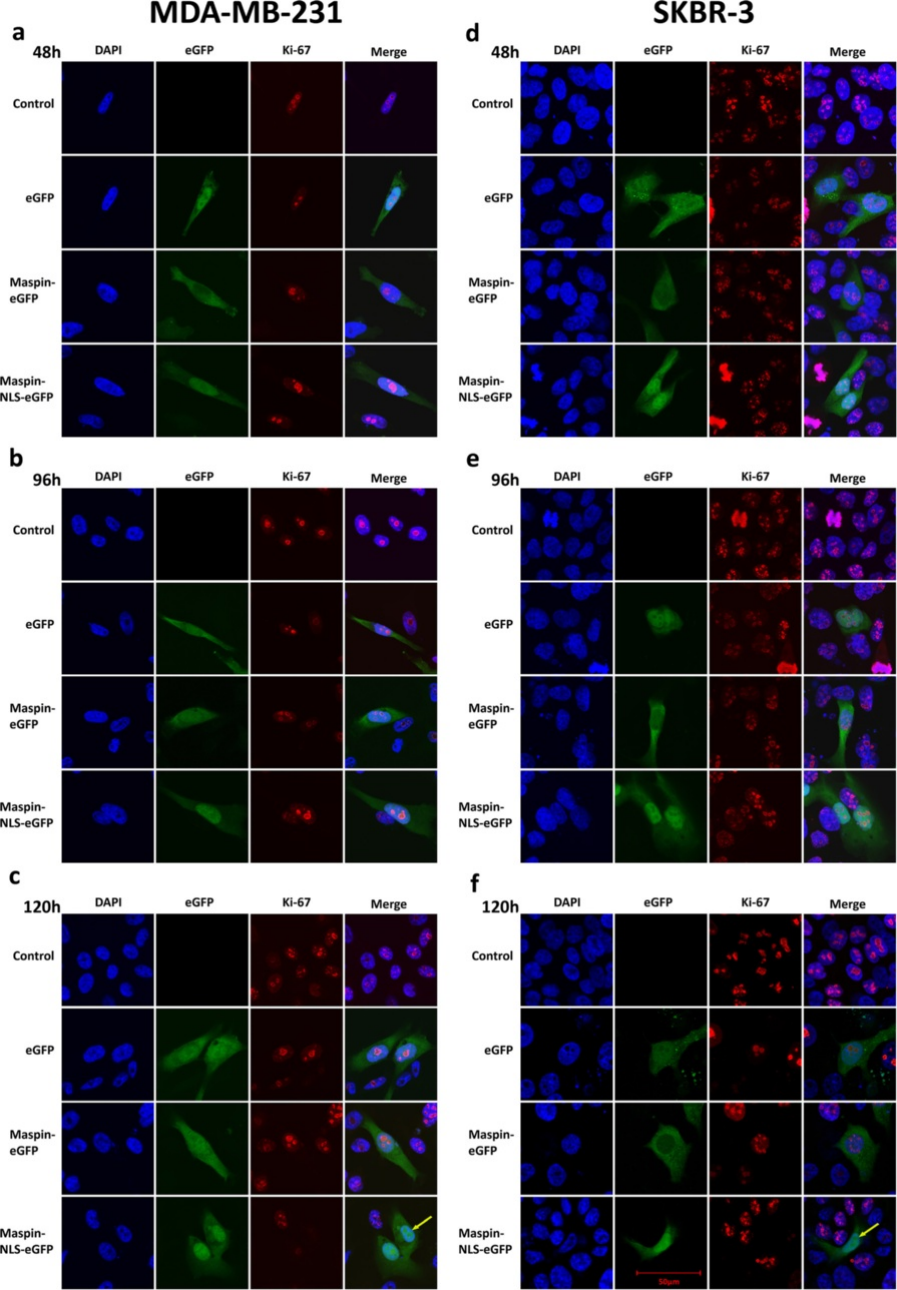
**Analysis of Ki-67 protein expression in MDA-MB-231 and SKBR-3 cells with various localization of maspin.** Cells were transiently transfected with plasmids EGFP, maspin-EGFP and maspin-NLS-EGFP and fixed 24 to 120 hours later; **a, b, d, e** - the majority of cells transfected with all three constructs express Ki-67 protein at 48 h and 96 h after transfection; **c, f** - the majority of cells expressing nucleus-localized maspin do not express Ki-67 at 120 h after transfection; simultaneously there is no such observation in control cells and cells expressing EGFP and msp-EGFP localized in cytoplasm; arrows show cells expressing nuclear maspin that lost Ki-67 protein.

In order to analyze the potential benefits for use of nuclear maspin as an anti-cancer genetic drug we decided to use additional breast cancer cell lines. Up to now we have tested the effect of maspin on MCF-7 breast cancer cell line (ER+, PR+, HER2 -). For our next studies we have chosen SKBR-3 cell line (ER-, PR-, HER2 +) and the highly aggressive, triple negative MDA-MB-231 cell line (ER-, PR-, HER2 -) [36].In transfected cells, there were no differences in Ki-67 staining pattern in comparison to control cells (Figure 6) and to the data gathered using MCF-7 cells (Figure 4). Nuclei morphology was not altered in transfected cells, but in some cases of cells expressing high levels of nuclear maspin, the cell nucleus was larger than in non-transfected cells. This may indicate polyploidy of these cells. After 48 h and 72 h, most cells that lost Ki-67 expression revealed a high level of maspin in the nucleus (Figure 6).The analyses of cells revealed, similarly to MCF-7 studies, that only nuclear maspin induces the loss of Ki-67 protein expression (Figure 6c,f). The majority of control, EGFP and maspin-EGFP cells in both cell lines expressed Ki-67 from 24 h to 120 h after transfection and the Ki-67 protein staining pattern in these cells was similar to that of MCF-7 cells. It seems that in SKBR-3 cells and MDA-MB-231 cells, the loss of Ki-67 expression in cells with nuclear maspin occurs later after transfection compared to MCF-7. In MCF-7 cell line, a decrease in Ki-67-positive cells expressing nuclear maspin was observed at 72 h after transfection, while in SKBR-3 and MDA-MB-231 cell lines gradual loss of Ki-67 expression was noted after 96 h.

### Nuclear maspin does not affect proliferation of normal epithelial cells

**Figure 7:**
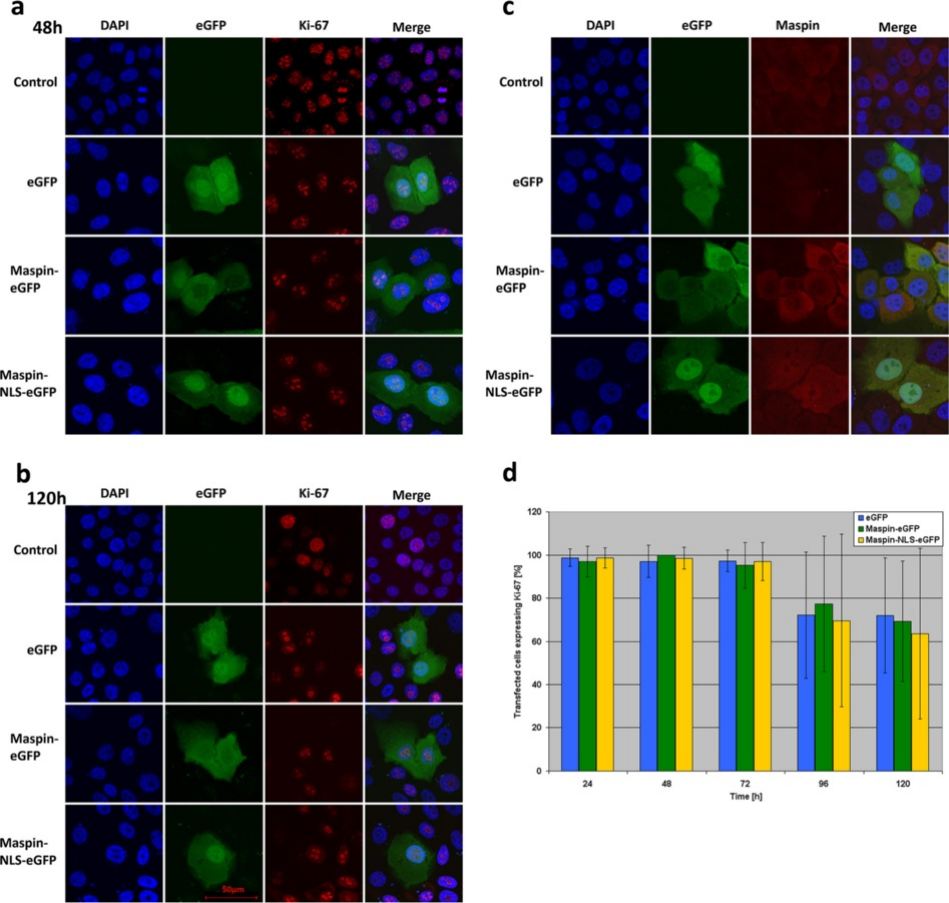
**Ki-67 protein expression in MCF10A cells with various localization of maspin.** Cells were transfected with plasmids EGFP, maspin-EGFP and maspin-NLS-EGFP and fixed after 24 to 120 hours; **a, b** - the majority of cells transfected with all three constructs express Ki-67 protein at 48 h and 120 h after transfection; **c** - maspin localization in MCF10A cells after transfection; MCF10A cell line expresses endogenous maspin which is mostly localized in cytoplasm; **d** - statistical analysis of Ki-67 expression in MCF10A cells transfected with three plasmids; there are no significant differences at 24 h to 120 h after transfection between cells expressing nucleus-localized maspin and those expressing both cytoplasm-localized maspin and EGFP.

The antiproliferative effect of nuclear maspin on breast cancer cell lines shows optimistic prospects for use of nuclear maspin as a potential genetic drug for breast cancer treatment. In order to test the effect of nuclear maspin on normal cell proliferation and potential unspecific cytotoxicity for normal breast cells we chose the epithelial normal cell line MCF10A. This cell line is ER-,PR- HER2- [36] and expresses maspin which locates more or less uniformly in the cytoplasm (Figure 7c).The level of expression of endogenous maspin in this cell line is several-fold lower than that achieved by transient expression of maspin using our constructs. Similarly to previous cell lines transfection of MCF10A cells with EGFP, maspin-EGFP and maspin-NLS-EGFP does not significantly affect the phenotype of cells. There is also no difference in number of Ki-67 protein-positive cells between control cells and those with EGFP, cytoplasmic maspin and nuclear maspin, especially during the first three days after transfection. After that time the number of Ki-67 protein positive cells varies in different transfections but changes are not statistically significant (Figure 7a, b and d). The decrease in number of Ki-67 protein-positive cells from 96 h to 120 h is the result of increased confluence of cells since normal epithelial cells are more susceptible to overpopulation and contact inhibition than breast cancer cells. We believe that these higher deviation errors arise from the contact inhibition since MCF10A control cells (data not shown) also show similar effects after 96 and 120 h.

The lack of inhibition of proliferation of MCF10A cells by maspin indicates that antiproliferative activity of nuclear maspin is restricted solely to/against breast cancer cells. This indicates that nuclear maspin may be used as a genetic drug in breast cancer treatment.

## Discussion

In many publications the various maspin localization has been confirmed, including the nuclear localization, which initially was ignored as an artifact [18,19,37]. Nuclear maspin localization was observed in breast, prostate, lung, colorectal, pancreas and larynx cancers [19,20,23-26,37].

Even if the anticancer effect of the nuclear fraction of maspin has been described in some reports, the precise significance of the maspin expression level and subcellular localization is still not completely understood. Moreover, recent reports on maspin function have focused on cytoplasmic maspin or undirected maspin introduction into the cells, which resulted in its cytoplasmic location [30,31,37]. Also the biological function of maspin and its influence on degree of differentiation, stage and invasiveness of cancer are not clearly established. Therefore we decided to investigate the effect of maspin localization on breast cancer with reference to clinical data from breast cancer specimens from patients (Figure 1).

Many reports have suggested that biological of maspin in various types of cancer depend on its subcellular localization [19-22]. Therefore, maspin protein level was evaluated independently in cytoplasm and nucleus in each specimen from patients.

In our study, a positive reaction to maspin was demonstrated in most specimens - 89.2%. A mixed reaction, localized both in nucleus and cytoplasm, was observed in most cases; only cytoplasmic localization was observed in 21.1% of cases and only nuclear in 13.3%. Similar data were obtained in studies in ovarian cancer, where most cases of low grading tumor exhibited maspin expression and most of them showed nuclear reaction [32]. In other studies on breast cancer specimens, about 96% of samples showed nuclear maspin expression and a cytoplasmic signal was present in 35% of the cases [19].

To evaluate the effect of maspin localization on cell proliferation, the proliferation status of cells in samples from patients was determined by immunohistochemical staining for Ki-67 protein (Figure 1j,k). The expression of proliferation marker Ki-67 is one of the main indicators of tumor cell proliferation and tumor grading. Moreover, it is used as a prognostic factor that helps to predict an outcome of cancer treatment [33,38]. Results obtained in our study indicated a statistically significant correlation between higher level of maspin protein in nuclei and decreased number of cells expressing Ki-67 (Figure 2c,d). These data suggest that the nuclear fraction of maspin has a strong influence on proliferation status of breast cancer cells in breast tissue, inhibiting their growth and division. The cytoplasmic fraction of maspin seems to have either an opposite effect than the nuclear pool or have no significant effect on proliferation status of breast cancer cells. Analyses of the Kaplan-Meier data indicated that in a time span of 2-5 years, lack of cytoplasmic maspin is beneficial over cytoplasmic maspin (Figure 2a,b). Also total lack of maspin is beneficial over low level of nuclear maspin (cumulative survival index 0.7 versus 0.2). The demonstrated correlation indicates the functional significance of each pool of maspin and suggests that maspin level and location may be considered for use as a prognostic marker as well.

To validate and compare results obtained in clinical studies, we decided to establish an *in vitro* model for assessing the functional significance of nuclear and cytoplasmic localization of maspin in breast cancer cell lines: MCF-7, MDA-MB-231 and SKBR-3. We also used normal epithelial cell line MCF10A as a control for normal breast tissue. These breast cancer cell lines do not express endogenous maspin because of its silencing mainly by epigenetic processes: CpG island methylation and histone deacetylation [39,40]. Control, normal cell line MCF10A does express maspin but at a relative moderate level and the protein is rather uniformly distributed in the cytoplasm.

All of the previously used methods of maspin re-expression or overexpression [32,41,42] in tumors and breast cancer cell lines had some disadvantages. Classical cDNA transfection, transduction by adenoviruses, protein administration and re-expression by artificial transcription factors resulted in cytoplasmic or extracellular localization of maspin. Such *in vitro* experimental models did not address properly the problem of dual maspin location observed in breast cancer tissues. That is why our major aim was to develop an easy and appropriate model for studies of the effect of differently located maspin fractions on breast cancer cell lines.

In order to devise a simple model system allowing for controlled placement of maspin in cytoplasm and cell nucleus, we used EGFP protein as a fusion tag. EGFP protein (27 kDa, no NLS and NES signals) alone is capable of two-way diffusion through nuclear pore complexes [43]. Our model allows for active transport of maspin with fusion protein but prevents free diffusion. For driving maspin-EGFP fusion protein into the cell nucleus we added a NLS signal sequence in a short linker protein fragment between maspin and EGFP (Figure 3b) to allow for proper folding of maspin and the EGFP part of the protein.Analyses of location of maspin fusion proteins in MCF-7 cells using confocal microscopy fully supported our idea of driving maspin fusion proteins to the cytoplasm and nucleus using EGFP and NLS (Figure 3c).In other breast cancer cell lines (SKBR-3 and MDA-MB-231) the expression level of maspin fusion protein was similar but the ratio between nuclear and cytoplasmic fractions of maspin NLS-EGFP fusion proteins was a little lower (lower efficiency of maspin placement to targeted location) (Figure 6). This effect may be caused by maspin retention or active transport by binding/interacting proteins. MCF10A cells show a similar ratio between the nuclear and cytoplasmic maspin protein (Figure 7c). When both endogenous and exogenous maspin is analyzed using antibodies against maspin protein the lower efficiency of maspin placement to the targeted location is achieved since endogenous maspin locates in cytoplasm (Figure 7c). Thus in transfected MCF10A cells the level of maspin protein in nuclei is comparable with all other cancer cell lines tested while cytoplasmic maspin (endogenous and exogenous together) is roughly about 10-20% higher than in tested cancer cell lines.

Taking the observations together, we successfully constructed a simple, efficient and complete model to study the influence of maspin localization on breast cancer cell growth, which can be used for further studies using transient transfection.Usefulness of this model was confirmed during our attempts to select cancer cell lines expressing nuclear maspin (maspin-NLS-EGFP), which failed completely and always within 2-3 weeks of selection. Since it was possible to select many cell lines expressing cytoplasmic maspin (Figure 3d) this demonstrated from the beginning the different and anti-proliferative activity of nuclear maspin. However, obtaining clones expressing maspin-EGFP required a longer period of time and lower concentration of selection antibiotic (because of cell death) than EGFP clones. Moreover, after transfection and a couple of days of selection, there were more EGFP-positive proliferating clones than in maspin-EGFP, despite the transfection efficiency being similar. Therefore, overexpression of maspin localized in cytoplasm may lead to slowdown of cell proliferation.

Nevertheless, there was no evidence that it was caused directly by a negative effect of cytoplasmic maspin on cells. It may be a result of overexpression of a larger protein with some biological functions, whilst EGFP is smaller and should not interfere with any cellular processes. However, previous reports demonstrated that exogenous maspin protein addition or transfection with plasmid encoding maspin (cytoplasmic location) resulted in increased cell sensitivity to cytotoxic agents, increased cell adhesion and inhibition of proliferation [42,44].

Proliferation assays and Ki-67 protein staining performed on MCF-7 breast cancer cell line indicated that the antiproliferative activity of maspin is the strongest in cells with nuclear maspin. For this reason, we have not been able to generate a stable breast cancer cell line expressing maspin localized in the nucleus and it does not allow us for precise studies of signaling pathways triggered by maspin. In the literature, there was no information about attempts of generation of a stable cell line with nuclear maspin, so it seems that this was the very first attempt to do so. Perhaps the generation of such a stable cell line will be possible only when maspin-NLS will be under a strictly controlled inducible promoter.Anti-proliferative activity of nuclear maspin on MCF-7 cells (Figures 4 and 5) was confirmed on other breast cancer cell lines: SKBR-3 cells and highly invasive MDA-MB-231 cell line (Figure 6). This indicates that nuclear maspin may be used as a prognostic marker and possibly as a therapeutic agent for cancer treatment using engineered maspin cDNA, for obtaining nuclear location of maspin, as a genetic drug in gene therapy.In order to confirm the potential usefulness of nuclear maspin as a genetic drug we tested its potential negative effect on normal breast tissue using normal epithelial cell line MCF10A. This normal breast tissue cell line expresses maspin which is located mostly in cytoplasm (Figure 7c). Transient expression of maspin did not have any significant effect on proliferation of MCF10A cells or the number of Ki-67 protein expressing cells (Figure 7a,b,d), thus confirming the high potential of nucleus-directed maspin as an anticancer agent in gene therapy. But still, the major question of what is the molecular mechanism of maspin action remains unanswered.

The major difficulty clouding the biological activity of maspin is lack of sufficient knowledge of molecular mechanisms regulating maspin subcellular location in normal and cancer tissues. In the maspin protein sequence, neither characteristic domains nor sequences which may target maspin to the nucleus were found. Passive transport to the nucleus is rather unlikely due to maspin’s weight (42 kDa) and structure. This is verified experimentally since simple expression of maspin cDNA in many cancer cells results in cytoplasmic location of the protein. EGFP (27 kDa) expression results in dual nucleo-cytoplasmic location.

Many proteins may be translocated to the nucleus as a result of phosphorylation, for example the transcription factors IRF (interferon regulatory factors) [45]. Maspin, in its structure, has many potential phosphorylation sites. *In vitro* studies on mammary epithelial cells transfected with maspin indicate that maspin protein is phosphorylated by a kinase domain from the epidermal growth factor receptor (EGFR) [46]. This may regulate interaction of maspin with its binding partners which in turn, depending on their function and modifications, may translocate to and from the cell nucleus, affecting maspin location. On the other hand, maspin presence in a particular fraction may modulate the activity of interacting protein.

Proteins that may directly interact with maspin are not well known, although some studies in recent years suggest direct interaction of maspin with proteins associated with oxidative stress (GST - glutathione S-transferase) [47], heat shock proteins (HSP), histone deacetylase (HDAC1-histone deacetylase I) [48], and IRF6 (interferon regulatory factor 6) [48], possibly indirectly affecting function of such transcription factors as Egr-1 and CGF2 [48]. Moreover, maspin may indirectly modulate the pro-apoptotic protein BAX (Bcl-2-associated X protein) [30], increase expression of the antiangiogenic thrombospondin and affect the expression of E2F1 [49,50]. Maspin is also implied in increase of expression of proteins from chromatin remodeling complex SMARCA2 and decreased expression of cytokines inducing inflammation and cell proliferation [49]. There was also observed maspin association with chromatin at the promoter of colony-stimulating factor-1 (CSF-1), which caused inhibition of tumor growth [22]. Some of these interactions may play an important role in maspin antiproliferation activity or maspin translocation to the cell nucleus.

The most interesting crosstalk between breast cancer cell proliferation and maspin offers two interactions of maspin: with IRF6 transcription factor and with HDAC1 in a GST-dependent manner. IRF6 promotes changes in cell adhesion, mobility and cell cycle probably (among others) through cadherin and vimentin. IRF6 locates both in cytoplasm and cell nucleus and interacts with maspin only when it is phosphorylated [51]. Recent studies demonstrated that IRF phosphorylation and subsequent proteasomal degradation are associated with induction of proliferation in normal breast epithelial cells (MCF10A). Concomitantly with this in breast cancer cell lines (MCF-7 and MDA-MD-231) overexpression of IRF6 together with maspin (located cytoplasmically) induced a synergistic effect of inhibition of proliferation but not total inhibition of proliferation [52]. This result resembles our data on proliferation slowdown by cytoplasmic maspin (Figure 5a,b).

The second directly interacting partner is HDAC1 complexed with GST. Cytoplasmic maspin may sequester complexes containing HDAC1 and GST and modulating its transport to nucleus and activity. Nuclear maspin may inhibit HDAC1 in a GST-dependent manner (oxidative stress sensitive) and prevent chromatin remodeling and change in transcription in an oxidative dependent manner [28].

The intriguing question is whether the loss of Ki-67 protein in cells with nuclear maspin is the cause or the result of the anti-proliferative effect of nuclear maspin on cells. The presence of Ki-67 protein is commonly used as a proliferation marker but its cellular functions are diverse and complex. During interphase it plays a crucial role in structural organization of nuclei, transcription and splicing of ribosomal RNA. The function of Ki-67 protein during mitosis is not fully understood but it belongs to the group of chromosomal passenger proteins and is located on mitotic chromosomes during mitosis [33,38]. Moreover, Ki-67 is necessary for forming a proper connection between microtubules and chromosome and for correct mitosis [53]. The time frame of cell death of nuclear maspin-containing cells fits exactly the 3-day interval between loss of Ki-67 protein (72 h) and cell death (144 h) (Figures 4, 5, and 6).

Exogenous and cytoplasmic maspin may increase cell adhesion by activation of ERK1/2 and MAP kinases and independently the PI3K pathway (through atypical PKCzeta) and inhibit cell mobility by inhibition of Rac1 and PAK1 pathways. Increased cell adhesion may cause inhibition of cell proliferation [31,54]. Therefore, overexpression of maspin localized in cytoplasm may lead to slowdown of cell proliferation, which may explain why breast cancer cells transfected with maspin-EGFP (cytoplasmic maspin) slow down proliferation but do not stop proliferating. Unfortunately, current knowledge of maspin does not provide any sufficient explanations for the function of nuclear maspin in complete blocking of proliferation of breast cancer cell lines in our model system. Since we also demonstrated that nuclear maspin is crucial for better prognosis and inhibition of proliferation of breast tumors *in vivo* it suggests that nuclear maspin may be successfully used as a prognostic marker and potential genetic drug for gene therapy for breast cancer.

## Conclusions

Our results indicate that nuclear maspin localization may be a prognostic factor in breast cancer and may have a strong therapeutic potential. High level of nuclear maspin is associated with better survival among breast cancer patients and lower proliferation status; thus nuclear maspin can be considered as a new marker of good prognosis of breast cancer. We also observed the inhibitory effect of nuclear maspin on cell proliferation *in vitro* in the three most frequently used breast cancer cell line models (MCF-7, SKBR-3 and MDA-MB-231), while there was no effect on proliferation of normal MCF10A breast cells. Maspin localization in the cell nucleus correlates with consecutive loss of Ki-67 protein, cell proliferation inhibition and cell death. Our data suggest a strong potential of nuclear maspin in breast cancer gene therapy treatment and provide a new insight into the role of maspin in breast cancer.

## Abbreviations

BAX: Bcl-2-associated X protein; CSF-1: Colony-stimulating factor-1; DAB: 3,3′-diaminobenzidine; DMEM: Dulbecco’s modified Eagle’s medium; EGFP: Enhanced Green Fluorescent Protein; EGFR: Epidermal growth factor receptor; Egr-1: Early growth response protein 1; EMEM: Eagle’s minimum essential medium; ER: Estrogen receptor; ERK1/2: Extracellular signal-regulated kinase 1/2; GCF2: GC-binding factor 2; GST: Glutathione S-transferase; HDAC1: Histone deacetylase 1; HER2: Human Epidermal Growth Factor Receptor 2; HSP: Heat shock proteins; IRF6: Interferon regulatory factor 6; Kip 1: Cyclin-dependent kinase inhibitor p27; NLS: Nuclear localization signal; NPCs: Nuclear pore complexes; PAK1: p21 protein activated kinase 1; PI3K: Phosphatidylinositide 3-kinases; PKCzeta: Protein kinase C isotype zeta; PR: Progesterone receptor; Rac1: Ras-related C3 botulinum toxin substrate 1; STAT3: Signal transducer and activator of transcription 3; WAP: Whey acidic protein.

## Competing interests

Ryszard Rzepecki, Miroslaw Sopel and Wrocław Medical University have applied for a patent entitled. “The polypeptide containing nucleus-directed maspin and its application in anticancer therapy” (Patent Application Serial number P.397195). MS has received salary from Wrocław Medical University during this study. We have no other competing interests to declare.

## Authors’ contributions

MM, KW, MS, and RR participated in designing of the study. MM, KW, MS and RR participated in data analysis and drafting the manuscript. MM and RR wrote the manuscript. MM generated stable cell lines, cultured cells, stained cells after transfection for immunofluorescence, performed the immunofluorescence analysis, carried out the cell proliferation assay, performed the statistical analysis. KW constructed plasmids, inserted NLS by mutagenesis, cultured cells, generated stable cell lines. MS performed IHC staining of samples from patients, evaluated maspin’s expression level and localization in these samples and provided statistical analysis of IHC results. RR supervised the study, participated in data analysis and interpretation of results and coordinate experiments. All authors read and approved the final version of the manuscript. All authors fulfill all requirements for authorship.

## Pre-publication history

The pre-publication history for this paper can be accessed here:

http://www.biomedcentral.com/1471-2407/14/142/prepub
